# Supplementary material for: Trafficking and Association of Plasmodium falciparum MC-2TM with the Maurer’s Clefts
Source: Pathogens. 2021 Apr 5;10(4):431. doi: 10.3390/pathogens10040431 (PMC8066109; doi:10.3390/pathogens10040431)
Supplement: Supplementary file 1 [file pathogens-10-00431-s001.pdf]

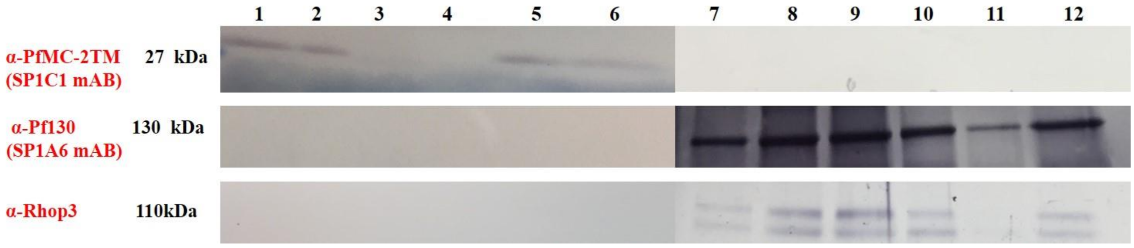

Figure S1. Immunoblotting analysis of differential extraction of PfMC-2TM from membrane ghosts. Lane 1, membrane ghost treated with 1% Triton X-100; lane 2, membrane ghost treated with 8M urea; lane 3, membrane ghost treated with 1mg/ml trypsin; lane 4, membrane ghost treated with 1% triton X-100 followed by 1mg/ml trypsin; lane 5, treated with 1M EDTA and lane 6, membrane ghost treated with 1M EDTA with freeze/thaw cycles. The lanes 7 to 12 are the supernatants from the respective extractions.

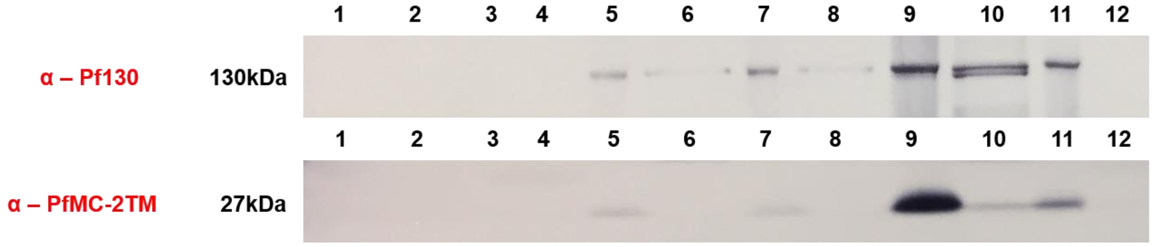

Figure S2. Membrane ghosts were subjected to alkaline sodium carbonate (Na2CO3) fractionation. Lane 1, uninfected RBC membrane ghost treated with Na2CO3 pellet; lane 2, uninfected RBC membrane ghost treated with Na2CO3 supernatant; lane 3, uninfected RBC membrane ghost treated with Na2CO3 and 1mg/ml trypsin pellet; lane 4, uninfected RBC membrane ghost treated with Na2CO3 and 1mg/ml trypsin supernatant, lane 5, infected RBC membrane ghost treated with Na2CO3 pellet; lane 6, infected RBC membrane ghost treated with Na2CO3 supernatant; lane 7, infected RBC membrane ghost freeze – thaw and treated with Na2CO3 pellet; lane 8, infected RBC membrane ghost freeze – thaw and treated with Na2CO3 supernatant; lane 9, Parasites treated with Na2CO3 pellet; lane 10, Parasites treated with Na2CO3 supernatant; lane 11, Parasites treated with Na2CO3 followed by trypsin digestion pellet and lane 12, Parasites treated with Na2CO3 followed by trypsin digestion (supernatant).
